# Understanding encroachment typologies through remote sensing and socio-economic analysis: enhancing national park management in Kerinci Seblat National Park, Indonesia

**DOI:** 10.1007/s00267-025-02218-x

**Published:** 2025-07-11

**Authors:** Muhammad Habib, Dedy Fitriawan, Kazuhiro Harada

**Affiliations:** 1 Graduate School of Bioagricultural Sciences, Nagoya University, Nagoya, Japan; 2 Vocational School: Remote Sensing and Geographic Information Systems, Universitas Negeri Padang, Padang, Indonesia

**Keywords:** Encroachment, Indigenous communities, National park management, Land use change, Sustainable livelihoods, Sustainable management

## Abstract

Encroachment remains a persistent challenge for Kerinci Seblat National Park (KSNP), despite its designation as a protected area. While agricultural use by local communities continues, limited understanding of the actors and their motivations hampers effective policy responses. This study addresses this gap by integrating remote sensing with socio-economic analysis to examine encroacher typologies and their spatial dynamics. Land use and land cover (LULC) changes were detected using Landsat 5, 7, and 8 imagery across six time periods (1988–2022), analyzed through maximum likelihood classification. Field surveys were conducted with 206 households, alongside in-depth interviews with customary leaders, village heads, and KSNP. We found an increasing trend of encroachment since the initial identification of the KSNP to the present, which correlates with the expansion of agricultural land. We grouped the typologies of encroachers into indigenous landless (23%), indigenous people with economic opportunities (29%), sly opportunists (2%), indigenous people as investors (3%), workers/profit-sharing partners (42%), and local migrants (1%). The dominant typology was workers/profit-sharing partners, which indicates that this partnership has a broad influence and wide coverage. Grouping actors supports the implementation of programs according to their motives and characteristics. The solution to encroachment should include a livelihood improvement program for indigenous people without land ownership, the establishment of utilization (e.g., agroforestry areas) and buffer zones, and enhanced law enforcement for other typologies.

## Introduction

Biodiversity conservation and the preservation of natural ecosystems are becoming increasingly important in the face of increasing anthropogenic activities and environmental changes (Izakovičová et al. 2022). Protected areas play a critical role in safeguarding natural treasures to ensure the survival of diverse species and maintain essential ecosystem services (Crofts and Gordon (2015)); however, the effectiveness of these protected areas is often hampered by factors such as encroachment (Atmadja et al. 2021), inadequate policy implementation, and insufficient monitoring (Kolahi et al. 2013).

Encroachment refers to unauthorized human activities in protected areas, including illegal logging, poaching, settlement expansion, and unregulated agricultural practices (Eden 1998; Saatchi et al. 2021). These activities pose significant threats to biodiversity and disrupt ecological balance (Corlett and Primack 2008). Various strategies have been implemented in different countries and regions to reduce forest encroachment. For instance, Brazil’s success in reducing deforestation rates is attributed to law enforcement, which involves key elements, such as the timely use of remote sensing technology to identify illegal deforestation, followed by the confiscation of timber and machinery (Tacconi et al. 2019). Another strategy is through education. Freund et al. (2020) found that environmental education programs have a positive impact on enhancing students’ knowledge and attitudes toward forest conservation and encouraging better government policies (Trewhella et al. 2005). In Indonesia, non-governmental organizations (NGO) have developed a strategy to reduce encroachment activities within national parks by improving livelihoods. This approach has been effective for households participating in the program but has yet to reach all encroachers (Habib and Harada 2023).

The formulation and implementation of targeted programs and policies are essential for the effective management of protected areas (Borrini-Feyerabend et al. 2013). Such efforts can help address specific threats, ensure the involvement of local communities, and promote sustainable practices that align conservation objectives with socioeconomic benefits (Harada et al. 2022; Gunawan et al. 2022; Sayer and Campbell 2004). Therefore, it is crucial to understand prevailing conditions in protected area ecosystems to make informed decisions regarding appropriate policies and programs (Watson et al. 2014). This involves regular ecological assessments to evaluate habitat health, species populations, and the extent of anthropogenic pressure (Hughes 2019; Leclère et al. 2020; Maxwell et al. 2020). Adaptive management strategies based on these assessments enable timely responses to emerging threats and changes in environmental conditions (Nichols and Williams 2006; Nichols et al. (2019)). Maintaining up-to-date knowledge of existing conditions ensures that conservation efforts are relevant and effective (Geijzendorffer et al. 2016).

Continuous evaluation and monitoring are crucial for measuring the success of conservation actions and understanding their impact on protected areas (Stem et al. 2005). This process involves setting clear success indicators and utilizing advanced monitoring technologies such as sensors, camera traps, and satellite imagery (Tacconi et al. 2019; Turner et al. 2015). Effective evaluation and monitoring not only track progress, but also identify areas needing improvement, ensuring that conservation strategies remain dynamic and responsive to changing conditions (Mace et al. 2018; Nichols and Williams 2006).

In Indonesia, encroachment in protected areas has become a serious issue as evidenced by significant rates of forest cover loss and land-use conversion. According to data from the Indonesian Ministry of Environment and Forestry (MoEF), between 2013 and 2020, more than 1.2 million hectares of forest within conservation areas were degraded due to illegal logging, encroachment, and agricultural expansion. Encroachment cases occur in various protected areas, including the KSNP, which is a United Nations Educational, Scientific and Cultural Organization (UNESCO) World Heritage Site. Typically, encroached land is converted into agricultural areas. Other issues include migration into the KSNP, road construction, illegal logging, mining, and geothermal energy development (Indonesian Ministry of Environment and Forestry, (2018)). Despite various efforts to address these issues, including the development of the Integrated Conservation and Development Project (1996–2002) through conservation agreements with the villages surrounding the KSNP, deforestation rates have not declined. (Linkie et al. 2008). Recently, a pilot program using an economic approach has been reported to reduce activities within the KSNP (Habib and Harada 2023); however, the project’s reach still needs broader expansion.

Relatively few studies have examined encroachers’ motives and activities in detail and linked them with encroachment rate data. For instance, Dwiyahreni et al. (2021) focused solely on the rate of forest loss without exploring its underlying causes in depth. Therefore, it is important to analyze encroachment levels across different periods (before and after the park designation). Thus, updated data on deforestation and encroacher identification is needed for more precise policy evaluation and monitoring. This study aimed to provide an essential framework for understanding the diverse roles and responsibilities of encroachers in the KSNP by linking land cover changes using satellite imagery interpretation with socio-economic motivations. This typology is fundamental for addressing the complex socio-environmental challenges associated with encroachment and can inform targeted conservation and resource management strategies within the park’s unique ecological context. This study analyzed encroachment rates from 1988 (before the establishment of the KSNP) to 2022 and identified encroaching households within the national park.

## Study site overview

The KSNP is the largest terrestrial conservation area in Indonesia, spanning 1,389,509 ha across four provinces: West Sumatra, Jambi, Bengkulu, and South Sumatra. It is also the largest national park in Southeast Asia and is designated as an Association of Southeast Asian Nations (ASEAN) Heritage Park. The KSNP plays a critical role in regional biodiversity conservation and safeguards economically significant rivers, including the Batang Hari River, which flows into Jambi Province. This study focuses on the upper watershed of the Batang Hari River, which is situated within the Kerinci Forest Management Unit and bordered by the KSNP in the Kerinci District. This upper watershed area forms the headwaters of the Batang Tebo River, a crucial tributary of the Batang Hari River. The KSNP region comprises diverse ecosystems, including montane highlands, lowlands, and swamp forests, with upland forests along the Bukit Barisan mountain range and lowland forests extending eastward toward the coast. The KSNP is also a vital habitat for numerous protected species and is instrumental in the conservation of endangered mammals, such as the Sumatran rhinocero (*Dicerorhinus sumatrensis*), Sumatran tiger (*Panthera tigris sumatrae*), Sumatran elephant (*Elephas maximus sumatranus*), siamang (*Symphalangus syndactylus*), Malayan tapir (*Tapirus indicus*), and Sumatran hare (*Nesolagus netscheri*). Additionally, the KSNP supports 524 plant species, including 126 orchids, 26 rattans, and 15 bamboo species. Notably, the park is home to the world’s largest flower, *Rafflesia sp*. (Indonesian Ministry of Environment and Forestry, (2021).

**Fig. 1:**
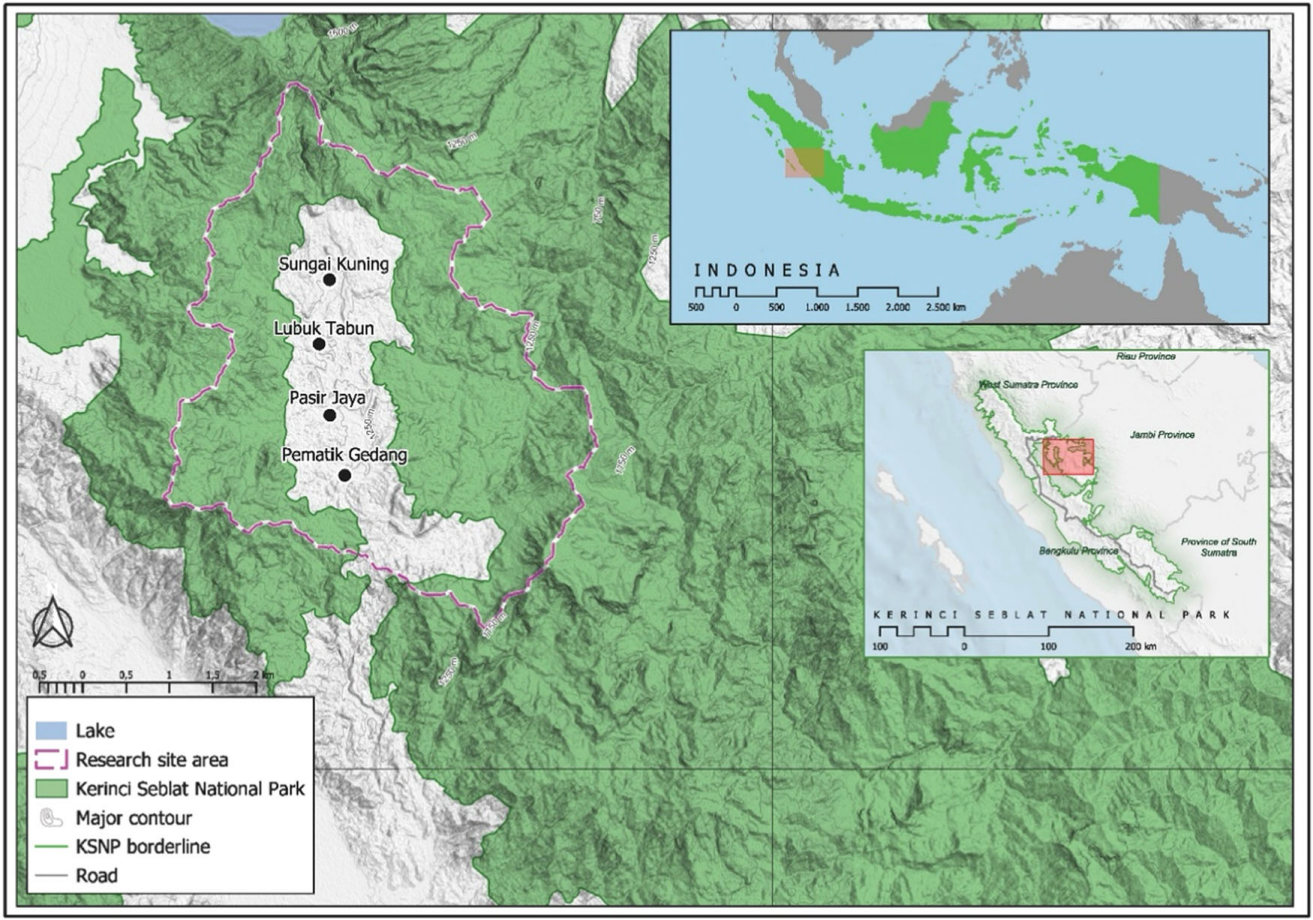
Study site

Adjacent forests are classified as production forests (PF), such as the Renah Pemetik region (Fig. 1), which is designated as a PF in the provincial spatial plan. The area was officially recognized as Forest Management Unit (KPHP) Unit I Kerinci based on Decree No. SK.960/Menhut-II/2013, which was issued by the Indonesian Minister of Forestry on December 27, 2013. The region covers an area of ~34,250 ha. The enclave includes three villages (Sungai Kuning, Lubuk Tabun, and Pasir Jaya) and one agricultural area (Pemetik Gedang). Altitudes in this area range from 1200 to 2100 m above sea level (masl) and encompass both alluvial plains and hilly Semangko Fault morphology. The annual rainfall ranges from 2700 to 3800 mm (Meteorological, Climatological, and Geophysical Agency, Jambi Province, 2023).

Local communities consist primarily of the Siulak indigenous people living in Sungai Kuning and Pasir Jaya, Semerup indigenous group in Lubuk Tabun, and Kemantan group in Pemetik Gedang. A significant portion of these indigenous communities engage in encroachment activities within the KSNP, with their primary livelihoods rooted in cultivating robusta coffee, cinnamon, and irrigated paddy rice. Local migrants from other provinces also live around and in the park and engage in encroachment, cultivating coffee and cinnamon as their main sources of income.

The KSNP is managed by the National Park Authority reports directly to the general directorate of natural resources and ecosystems conservation under the Indonesian Ministry of Environment and Forestry. Any program to be implemented within the park’s boundaries must obtain permission and collaborate with the park authority. This also applies to local and international NGOs working toward the sustainability of the KSNP, including organizations such as Flora and Fauna International (FFI), the World Wildlife Fund (WWF), and local NGOs such as Lembaga Tumbuh Alami (LTA). One notable LTA program involves the cultivation of arabica coffee by local communities living around the national park. Farmers are provided with arabica coffee seedlings and shade trees to plant on designated land outside the national park using an agroforestry system (Habib and Harada 2023). This program serves as an indicator for assessing trends in encroachment before and after the program implemented.

## Methodology and analysis

### Processing of Landsat satellite imagery for land use land cover change

This study analyzed the changes in the KSNP’s forest and land cover using Landsat imagery from 1988 to 2022 at a spatial resolution of 30 m. The dataset included images from Landsat 5 (TM) for 1988, 1990, 1994, and 2006, and Landsat 8 (OLI-TIRS) for 2014 and 2022 (Table 1). To ensure accuracy, we used cloud-free Landsat images for data collection. Images fully or partially obscured by clouds were excluded to prevent inaccuracies. The downloaded images were processed using ENVI version 5.1 software and produced outputs that were subsequently analyzed and visualized in ArcGIS Pro. This workflow ensured reliable data processing and interpretation for evaluating changes in land cover over the study period.

**Table 1:** Summary of the collected Landsat data

Path/row	Dataset	Res.	Entity ID	Date acquired
126/61	Landsat 5 TM	30 m	LT05_L1TP_126061_19880613_20200917_02_T1	1988/10/03
126/61	Landsat 5 TM	30 m	LT05_L1TP_126061_19900603_20200915_02_T1	1990/06/03
126/61	Landsat 5 TM	30 m	LT05_L1TP_126061_19940411_20200913_02_T1	1994/04/11
126/61	Landsat 5 TM	30 m	LT05_L1TP_126061_20060530_20200901_02_T1	2006/05/30
126/61	Landsat 8 OLI_TIRS	30 m	LC08_L1TP_126061_20140621_20200911_02_T1	2014/06/21
126/61	Landsat 8 OLI_TIRS	30 m	LC08_L1TP_126061_20220526_20220602_02_T1	2022/05/26

United States Geological Survey (USGS)

Geometric corrections were performed on all images to align them with the UTM Zone 47S map projection and WGS84 Datum. The average root mean square error (RMSE) was less than 0.5, indicating high geometric accuracy. Ancillary data, including ground truth data obtained through field visits, were incorporated to validate the land use and land cover (LULC) classifications.

Nine LULC classes were defined for the study area: (1) bare land, (2) forest, (3) paddy field, (4) grass, (5) seasonal crops, (6) annual crops, (7) bush and shrub, (8) settlement, and (9) water body. A visual interpretation of the various band combinations was utilized to select training samples for each LULC class, which were subsequently verified using ground truth points and high-resolution satellite imagery. A signature file was created from the training samples containing multivariate statistics for each LULC class. These training samples were used as inputs for the maximum likelihood classification (MLC) method, a widely applied supervised classification technique (Hassan et al. 2016). MLC, combined with a post-classification comparison (Allam et al. 2019), involves classifying each image and generating thematic maps. These maps are then compared pixel by pixel between two dates to detect changes. The resulting changes provide insights into community pressure on land and trends in encroachment, offering a detailed understanding of the dynamics affecting the study area.

To assess the reliability of the classification results, an accuracy assessment was conducted using a stratified random sampling approach. A total of 450 sample points were proportionally distributed across the nine LULC classes, with a minimum of 10 samples per class, irrespective of class area—following best practice guidelines outlined by Olofsson et al. (2014). Reference data were derived from expert-based visual interpretation of high-resolution satellite imagery (2022–2023) available through Google Earth Pro. Each sample point was labeled based on its visual correspondence with the classified image; where discrepancies were found, the correct land cover class was assigned and used to construct the confusion matrix. From this matrix, standard classification accuracy metrics were calculated, including Producer’s Accuracy (PA), User’s Accuracy (UA), Overall Accuracy (OA), and Cohen’s Kappa coefficient. These metrics were used to evaluate the robustness and consistency of the classification, ensuring its suitability for subsequent spatial analysis.

### Data collection through fieldwork

The field survey was conditioned as follows: (1) Field observations were done by direct observation of the location (community-based village resource inventory). While conducting observations, both individual and institution/group information sources were identified before subsequent surveys. (2) Focus group discussions (FGDs) and in-depth interviews were conducted to gather qualitative data. Two FGDs were conducted, which involved 17 participants, consisting of 14 men and three women. In addition, 13 in-depth interviews were conducted with nine men and four women. FGDs and interviews were conducted with the relevant stakeholders, namely KSNP officials, NGOs, *adat* leaders, and indigenous people. (3) Household surveys were conducted, and key respondents were identified. The survey was conducted in July–August 2022 and the number of target respondents was 206 households out of the 654 identified as residing within the national park, based on data provided by the local NGO, Lembaga Tumbuh Alami (LTA). The respondent was collected using haphazard sampling, with consideration given to the spatial distribution of households across encroachment areas.

### Data analysis

**Fig. 2:**
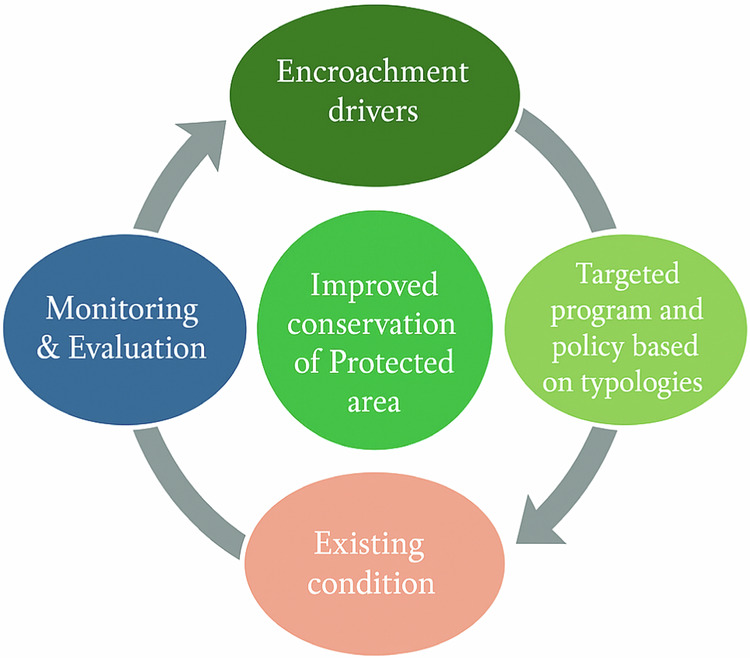
Logical framework of the study

This study used a combination of qualitative (descriptive analysis, field data recap, and thematic analysis) and quantitative (data tabulation, graphical representation, and statistical analysis) methods to analyze the various types of data, including field data, household survey data, and program outcomes. Map and image data were processed using ArcGIS or similar methods and were presented in the form of maps and data. While the results of the other field data were tabulated, the recap and analysis of the results were displayed in the form of tables, graphs, diagrams, and percentages. Sample households were selected for the survey based on the encroachment map. We analyzed the household survey data to classify encroacher typologies based on factors such as origin, arrival in the area, motives, type of farming or business activities, and land acquisition methods. The results of the map and trend LULC, household survey, and FGDs with key informants were analyzed based on the logical framework (Fig. 2). The primary objective of the framework is to support improved conservation outcomes in the PA by integrating a theory of change approach to better illustrate the causal relationships, key processes, and feedback mechanisms involved in addressing encroachment in KSNP. This approach is structured as a cycle of interconnected components: encroachment drivers, this component identifies the socio-economic, cultural, and institutional factors that drive land-use change and illegal occupation. Targeted programs and policies, based on encroacher typologies identified through household surveys and spatial analysis, tailored interventions are proposed. Existing conditions, field data from LULC analysis, household surveys, and interviews provide insights into current encroachment patterns and community dynamics, informing both typology development and the feasibility of interventions. Monitoring and evaluation, monitoring is conducted first to observe changes, followed by evaluation to assess the effectiveness of interventions and adjust policies accordingly. This cyclical feedback loop ensures iterative learning in response to dynamic human-environment interactions and strengthens adaptive management in conservation planning. We focused on the current conditions in encroached areas and examined encroachment trends during the following key periods: national park identification process (1988), start of boundary surveys (1990), completion of boundary surveys (1994), as well as before (2014) and after (2022) the encroachment control project based on the arabica coffee planting program. Encroachment typology data were used to assess field conditions, providing essential insights for evaluating and recommending improved management policies.

## Results

### Encroachment trends before the establishment of the KSNP and after the program’s implementation

In 1982, during the Third World National Parks Congress in Bali, the Indonesian Minister of Agriculture announced the designation of the area now known as the KSNP as a proposed national park (Indonesian Ministry of Environment and Forestry (2018)). Following this declaration, research projects were initiated to explore the area’s biodiversity and to design the park’s boundaries. In the 1990s, formal efforts to delineate and establish the boundaries of the KSNP began, including activities at the Kerinci site. The region known as Renah Pemetik was initially designated for inclusion in the national park. Interviews with key figures during the designation period indicate that the government at that time had requested the indigenous communities within this area to relocate; subsequently, a collaborative boundary mapping process was conducted involving the local community, government authorities, and the WWF. This process ultimately led to the exclusion of a substantial portion of land previously occupied by the local community from the designated national park, allowing these areas to remain outside the protected zone.

**Fig. 3:**
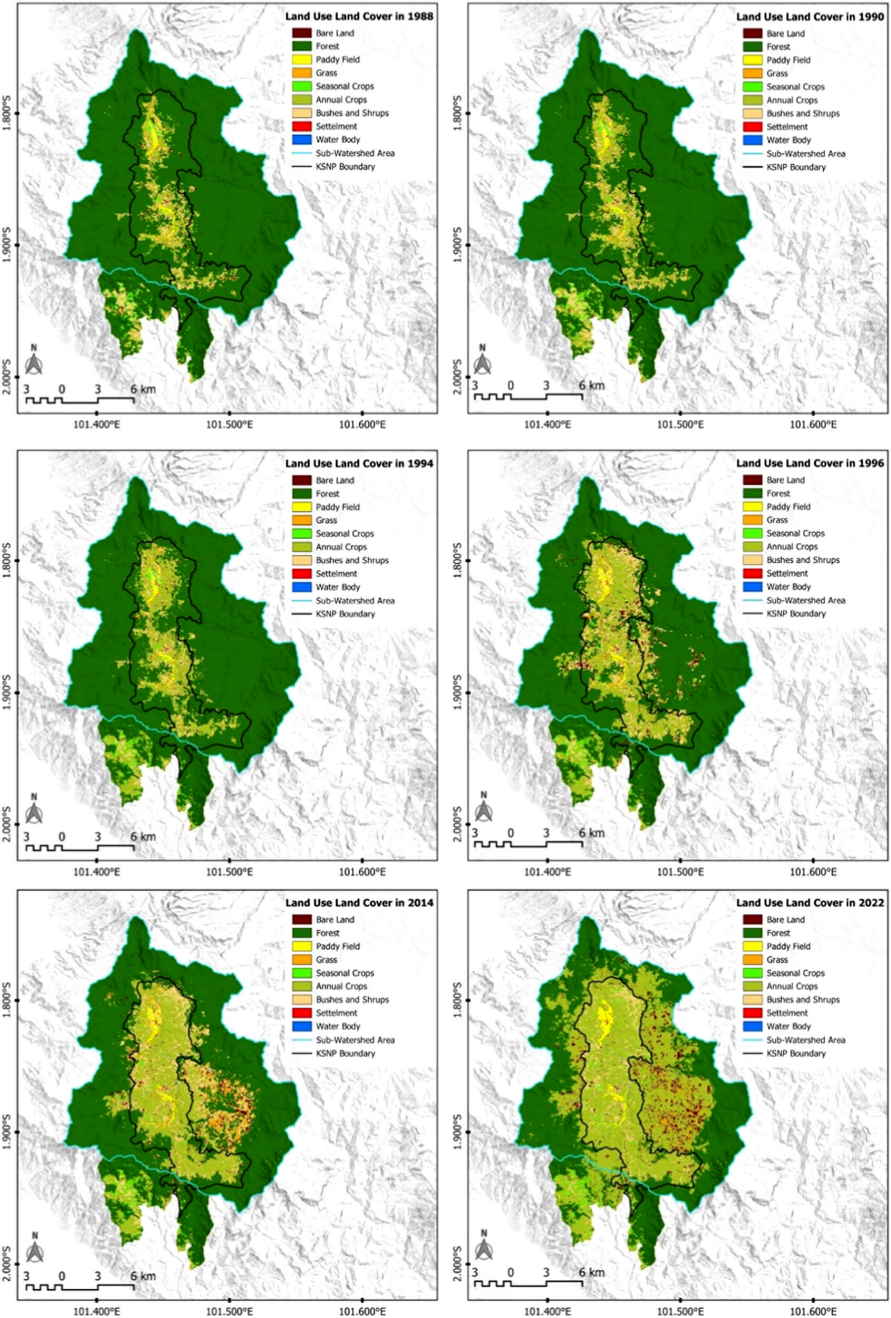
Changes in land use and land cover (1998–2022)

We identified changes from forested to non-forested areas by overlaying the established boundary map with land cover data before the park’s designation (1988), during the boundary mapping process (1990), and after the measurements were finalized (1994), as shown in Fig. 3.

The results of our surveys confirm that a portion of the local population engaged in agricultural activities within the designated national park area prior to its official establishment. Although these communities occupied the area before formal designation, they are legally classified as encroachers. Encroachment areas have expanded over time, driven by changes in land use and increasing migration into these enclaves (Table 2).

**Table 2:** Household migration to NP

Period	Number of households have moved
<1990	7
1990–1995	34
1996–2000	25
2001–2010	69
2011–2020	62
>2020	9
Total	206

Field survey 2022

**Fig. 4:**
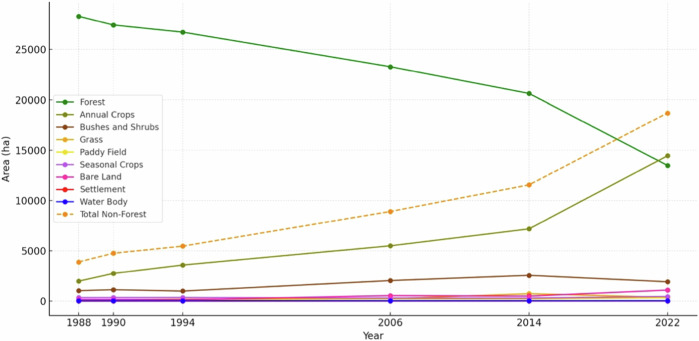
LULC trends (1988–2022)

Indigenous communities had already settled in these enclaves before boundary measurements and the formal establishment of the KSNP. Satellite imagery reveals substantial changes in land cover from 1994 to 2022 (see Fig. 4). This aligns with the increasing number of households migrating into the national park area, along with the desire of already-settled communities to expand their cultivation areas, as shown in Table 2. This condition has contributed to the sharp increase in deforestation over the past decade. The predominant transformation from forest to non-forest cover is attributed to the cultivation of annual crops, such as robusta coffee and cinnamon plantations, with cinnamon being the primary crop. Robusta coffee is initially planted to facilitate cinnamon growth, which typically reaches a height of 3–4 m and matures within 4–5 years.

### Typology of encroachers in the KSNP

#### Duration of encroachment activities

The primary indicator for assessing encroacher typology is the timing of their presence within the national park. We categorized encroachers into two groups: those who were present before the park’s official designation and those who arrived after the park was designated. Our analysis shows that 20% of the respondents were already residing within the park’s boundaries prior to its official designation, while the remaining 80% arrived after the park had been formally established (Table 2). The second indicator is the status and role of households in the park.

#### Role and status of households

The status and role of households within the KSNP reveal four distinct and influential categories: landlord, landlord and cultivator, sharecropper, and worker, each contributing uniquely to the complex dynamics of land use and resource management within the park. As summarized in Table 3.

**Table 3:** Status and role of households within the KSNP

Category	Main role	Primary goal	Typical activities
Landlord	Owns land; acts as facilitator and financier, not directly involved in farming	Facilitate agricultural operations and earn income through partnerships	Providing capital and land, managing partnerships
Landlord and Cultivator	Owns and cultivates land; collaborates with sharecroppers due to limited capacity or land	Secure livelihood and optimize land use through cooperation	Farming own land, coordinating with sharecroppers or landlords
Sharecropper	Partners with landlords; actively involved in farming and resource sharing	Secure main source of livelihood through cooperative farming	Farming, sharing inputs and outputs with landlords
Worker	No land ownership; works as labor to support farming activities	Earn income through labor; secure livelihood without owning land	Land clearing, harvesting, transporting crops (coffee and cinnamon)

**Fig. 5:**
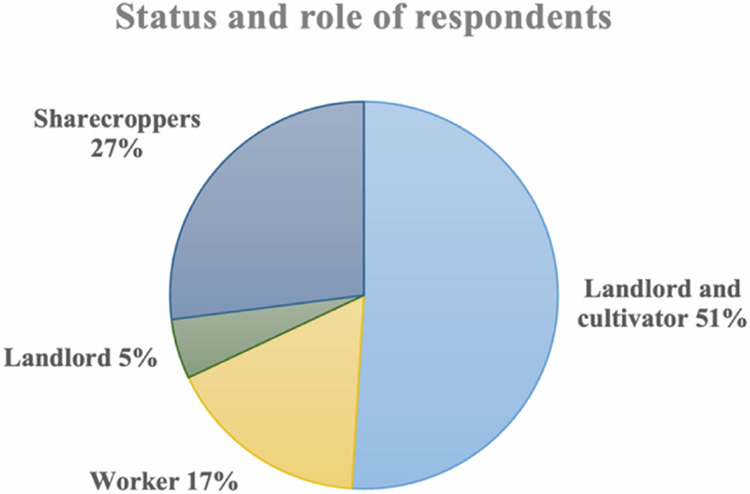
Household categories in the KSNP

Landlords are individuals who own the land but primarily serve as facilitators and financiers in cooperative arrangements. Rather than engaging directly in farming activities, landlords collaborate with other farmers, using their land as leverage to facilitate agricultural operations. This group represented 5% of the respondents (Fig. 5). It consist of indigenous communities and local migrants; most are entrepreneurs or government employees.

The landlord and cultivator group comprised 51% of the total respondents. The primary goal of landlords and cultivators is to secure a livelihood. In addition to actively managing their own land, they collaborate with sharecroppers to cultivate their land because they often lack the capacity to manage their land effectively. Some also partner with other landlords due to their limited amount of land to generate additional income.

Sharecroppers partner with the landlords and participate in shared farming activities and resource allocation. Operating within a cooperative framework, they work jointly with the landlords to tend to the land and share in the resulting benefits. In this study, 27% of the respondents were sharecroppers. Sharecroppers’ primary goal is to secure their primary source of livelihood. Some households also work as laborers on other people’s land to supplement their income, particularly outside the harvest season (robusta coffee).

The worker group includes individuals who make significant contributions to land utilization in the park despite lacking ownership rights. Workers perform essential tasks, such as land clearing, crop harvesting (particularly coffee and cinnamon), and transporting produce for sale, making their labor integral to productivity and economic activities within the park. The primary objective of this group is to secure a livelihood as they do not have land to cultivate independently. Additionally, they require landlords who are willing to offer collaboration. In this study, workers represented 17% of the respondents.

The collaborative framework of landlords and sharecroppers in the KSNP reveal a rich tradition within the context of encroachment. Although often characterized by informality, our research has unearthed several written agreements that encapsulate the essence of these partnerships, where the agreements provide intricate details of the key elements of cooperation such as the land’s scope, provision of resources, and an equitable division of harvests. This study identified several distinct types of agreements between landlords and sharecroppers (Table 4), each reflecting varying levels of resource provision and harvest-sharing mechanisms.

**Table 4:** Summary of sharecropping agreement types

Agreement type	Support provided by landlord	Harvest sharing arrangement
Comprehensive support agreement	- Coffee and cinnamon seeds -15 kg of rice per month for 2 years - Rp. 1,500,000 per month	- Shared equally - Only for coffee
Three-part division agreement	- Coffee and cinnamon seeds - 15 kg of rice per month for 2 years	- Divided into three parts: 1 part to landlord and 2 parts among sharecroppers
Substantial initial support agreement	- Coffee and cinnamon seeds - One-time contribution of 150 kg of rice	- Harvest split equally between coffee and cinnamon
Limited support agreement	- Coffee and cinnamon seeds	- Sharecroppers retain full coffee harvest - Cinnamon harvest shared equally
No provision agreement	- No resources provided	- After first coffee harvest, land is divided equally between landlord and sharecropper

Local communities commonly adopt cooperative systems such as the comprehensive support agreement and the three-part division agreement, as these arrangements are perceived to be more mutually beneficial for both parties. Under these agreements, sharecroppers have better assurance that their daily needs will be met during the pre-harvest period, while landlords tend to rely on the long-term investment value of land and cinnamon gardens, which do not require profit-sharing with the sharecroppers. These agreements illustrate the diverse roles and responsibilities of land utilization within the KSNP, emphasizing how different levels of landlord involvement shape resource allocation and harvest-sharing practices.

#### Land acquisition methods

**Fig. 6:**
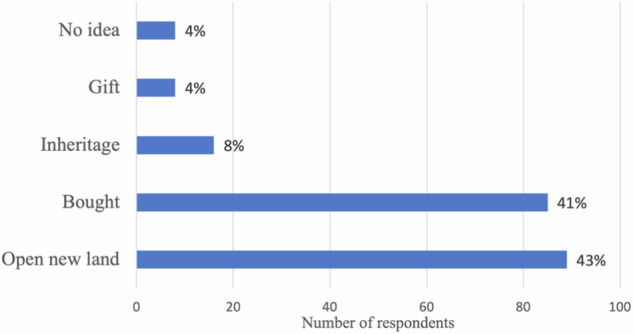
Land acquisition methods

The majority of land encroachers acquire their land through either direct purchase or forest-clearing. As shown in Fig. 6, 43% of the respondents cleared forests to obtain land, while 41% said they acquired land through purchase agreements. This indicates the presence of land transactions and land speculation within the national park.

Some land encroachers said they received their land as a gift, either as compensation from landlords or as part of an agreement. Only 8% of the respondents said they obtained their land through family inheritance, which suggests second- or third-generation encroachment in the national park. Some respondents said they were unaware of the origin of the land they worked on as they are employed as laborers or engaged in sharecropping arrangements.

#### Types of cultivation or economic activities

The land cover change map shows that most forests are cleared for dryland agriculture (annual crops), which aligns with our data, since 88% of the respondents cultivated robusta coffee and cinnamon. This pattern reflects the traditional local practice of minimizing the maintenance costs of cinnamon cultivation. Only 7% of the farmers exclusively planted cinnamon. These farmers typically cultivate land previously harvested for cinnamon, where they only need to wait for shoots from old stumps to start growing. The remaining 5% of the respondents grew a mix of robusta coffee, cinnamon, and horticultural crops. The cultivated lands are generally located near settlements or villages and are easily accessible. This type of farming serves as households’ primary source of income. The size of the land cultivated by farmers varies, ranging from 1 ha to a maximum of 23 ha, with an average of 3.9 ha and a median of 3 ha per household.

#### Awareness of laws and law enforcement by KSNP officials

**Fig. 7:**
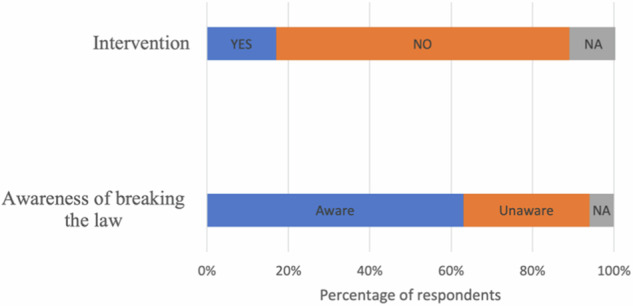
Respondents’ awareness of laws and law enforcement by KSNP officials

Under Indonesian law, encroachment activities in national parks are considered illegal. The legal basis for this is Law No. 32 of 2024 concerning the Conservation of Biological Natural Resources and Their Ecosystems, which replaced Law No. 5 of 1990 and introduced stricter regulations and enforcement mechanisms. While most respondents were aware that their activities within the KSNP constituted a breach of the law (Fig. 7), the majority surprisingly reported that they had never received any interventions or enforcement action.

The respondents’ statements indicate that they were aware of KSNP officials and law enforcement officers taking action to address encroachment activities. These actions included issuing warnings to prevent the clearing of new land and to vacate cultivated areas. In more severe cases, actions included evictions, the burning of farmhouses, destruction and cutting down of crops, and even arrests followed by legal proceedings. One of the most well-remembered cases among local communities is the arrest of five farmers during a joint operation conducted in 2012 by the national park authority, the police, and the military. Investigations revealed that these individuals were not landowners but were working as sharecroppers.

### Classification of respondents based on encroacher type

Based on the key characteristics mentioned in section 4.2.1–4.2.5, Table 5 classifies encroachers into six groups, each with distinct characteristics.

**Table 5:** Typology of encroachers and their characteristics

Encroachers	Characteristics	
Type 1		
Indigenous landless	Origin	Native to the area
	Arrival in the area	Mostly before the creation of the park (before 1996)
	Motive	Earn a livelihood
	Type of farming or business	Plantation crops (robusta coffee and cinnamon)
	Land acquisition method	Forest clearing or inheritance
Type 2		
Indigenous economic opportunity	Origin	Native to the area
	Arrival in the area	Mostly after the creation of the park
	Motive	Investment opportunities
	Type of farming or business	Plantation crops (robusta coffee and cinnamon)
	Land acquisition method	Land purchase and hiring workers for land cultivation
Type 3		
Sly opportunists	Origin	Native or migrant to the area
	Arrival in the area	After the creation of the park
	Motive	Financial/business, investment, land speculation, and political interest
	Type of farming or business	Land trading, forest clearing, and plantation crops (robusta coffee and cinnamon)
	Land acquisition method	Land purchase and forest clearing
Type 4		
Indigenous people as investors	Origin	Native to the area
	Arrival in the area	Before or after the creation of the park
	Motive	Investment and business opportunities
	Type of farming or business	Plantation crops (coffee and cinnamon)
	Land acquisition method	Hires other workers to clear new land
	Special feature	Employs many people with a profit-sharing system
Type 5		
Worker or profit-sharing partner	Origin	Native or migrant to the area
	Arrival in the area	Mostly after the creation of the park
	Motive	Earn a livelihood
	Type of farming or business	Plantation crops (robusta coffee and cinnamon)
	Land acquisition method	Cultivates land owned by Type 4; often does not own land
Type 6		
Local migrant	Origin	Another province
	Arrival in the area	After the creation of the park
	Motive	Earn a livelihood and economic opportunity
	Type of farming or business	Plantation crops (robusta coffee and cinnamon)
	Land acquisition method	Land purchase and forest clearing
	Special feature	Operates secretly, rarely interacts with locals, gains access through Type 3 encroachers (sly opportunists), settles in remote areas, and lives in groups

**Fig. 8:**
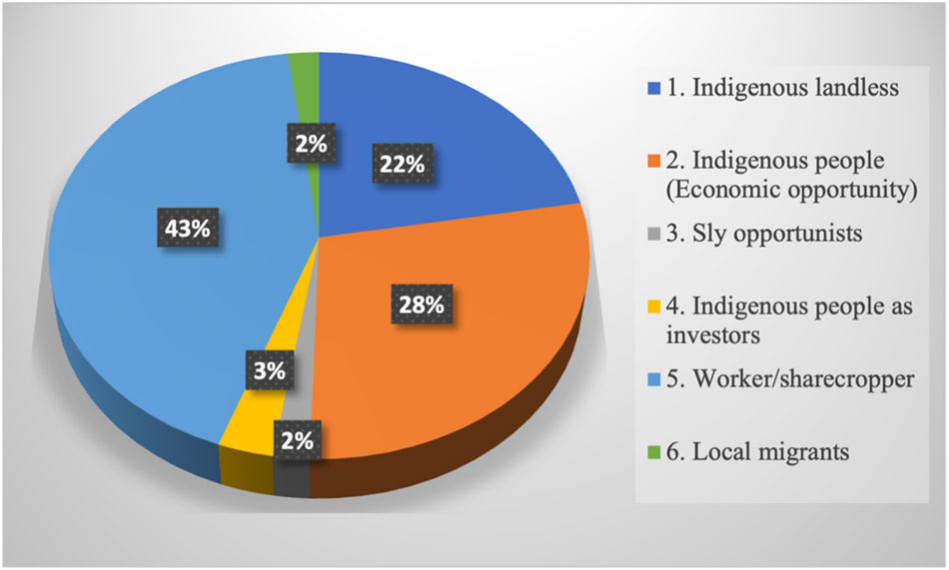
Proportion of encroacher typologies in the KSNP

We classified the respondents by encroacher typology (Table 5); the proportions are shown in Fig. 8. The majority of the respondents were workers or profit-sharing partners (42%), indicating that most were under the control of landlords, investors, or individuals engaged in opportunistic practices. The primary goal of the indigenous economic opportunity type (28%) was investment. Their main sources of income were often outside the national park, such as government employment or trade. In contrast, the indigenous landless type (22%) depended entirely on the KSNP for their livelihood since they had no land or alternative sources of income. The remaining respondents were categorized as sly opportunists (2%), indigenous investors (3%), or local migrants (3%), and were responsible for most of the encroachment in the KSNP. They were most evident in encroached areas and engaged in collaboration, land transactions, and other activities.

## Discussion

The sharp increase in the encroachment rate from 1994 to 2022 indicates a significant rise in cases following the designation of the area as a national park. Economic motives and weak monitoring by the relevant authorities are the primary drivers behind the encroaching activities in the KSNP. This finding aligns with those of Sunderlin and Resosudarmo (1996), who observed that encroachment tends to increase with the establishment of boundaries and national parks, coupled with rising demand and weak law enforcement and monitoring efforts. This finding is also consistent with the observations of Dwiyahreni et al. (2021), who reported that most national parks have experienced forest cover loss primarily due to human activities.

The conversion of forest cover to agricultural land is not a new phenomenon; however, a deeper analysis reveals diverse motivations for farming within the KSNP. The increasing trend of encroachment and the classification of encroachers serve as references for stakeholders to evaluate and monitor the effectiveness of programs. This aligns with the perspective of Nichols et al. (2019), who emphasized the need for adaptive management strategies and appropriate responses to emerging threats.

Generalizing encroachers and their solutions reduces the likelihood of success in preventing and mitigating encroachment. Borrini-Feyerabend et al. (2013) mentioned the importance of designing and implementing effective programs and policies for managing protected areas. Categorizing encroachers based on their motives and characteristics supports the implementation of tailored programs that align with the encroachers’ specific needs and motivations. Thus, we propose an adaptive strategy to address encroachment through the formulation and implementation of appropriate policies based on the typology of encroachers. This strategy is framed within a theory of change approach, outlining the expected pathways from policy interventions to behavioral shifts and ultimately, improved conservation outcomes as emphasized by Taplin et al. (2013).

First, the indigenous landless group’s primary motivation is subsistence farming to meet their basic needs. Their presence prior to the establishment of the KSNP’s boundaries highlights the need to reevaluate the boundary-setting process at the time of designation. Special policies are required to balance both conservation goals and livelihoods. Implementing economic improvement programs based on agroforestry systems increases the likelihood of achieving these dual objectives. As reported by Habib and Harada (2023), economic enhancement programs for encroachers outside the national park have successfully reduced their activities within the park. Similarly, Blouch (2010) emphasized that the establishment of traditional use or utilization zones with clearly defined boundaries could be effective for indigenous communities who were already residing within the national park prior to its designation. The opportunity for managing the KSNP in collaboration with indigenous communities is substantial, as demonstrated by their proven ability to manage customary forests in their villages. Collaboration should be aimed at sustaining their livelihoods and protecting their settlements from natural disasters (Harada et al. 2022). Similarly, other social forestry schemes such as community conservation agreements and collaborative forest management offer promising frameworks to integrate biodiversity conservation with participatory governance. These approaches, as emphasized by Gunawan et al. (2022), provide legal recognition for community stewardship, enhance local livelihoods, and support conflict resolution in protected areas. One of the agreements with indigenous communities involves collaboration in planting timber trees and taking responsibility for preventing and reporting new encroachers entering surrounding areas, which are practiced in Meru Betiri National Park (Nurrochmat et al. (2017), Harada et al. 2021).

Second, the indigenous economic opportunities group represents encroachers whose primary motivation is investment rather than fulfilling their basic needs. This group views cinnamon cultivation as a future investment opportunity, motivated by its consistently increasing market prices and low maintenance costs. Typically, they harvest cinnamon to cover significant expenses such as their children’s education, house construction, or religious pilgrimages such as Hajj. This group generally has a higher level of education, making them more receptive to outreach and educational programs. As discussed by Freund et al. (2020), training and education can have a positive impact on their knowledge and attitudes. Since some of the individuals in this group engage in land transactions with so-called sly opportunists, it is also necessary to strengthen monitoring and law enforcement to prevent further encroachment.

Third, indigenous investors, sly opportunists, and local migrants (8%) represent a small group of individuals who control most of the land within the national park, making them the primary contributors to encroachment. Addressing this group requires a strategy centered on law enforcement, incorporating key elements such as the timely use of remote sensing technology to detect illegal deforestation (Tacconi et al. 2019). The effective targeting of this group will also have a broader impact on other encroacher types, particularly sharecroppers and workers, who are heavily dependent on these dominant groups for access to land and resources. Strengthening enforcement measures against this group can disrupt the networks that facilitate further encroachment, reduce reliance, and prevent the expansion of illegal activities.

Furthermore, the implementation of all programs and policies must be followed by an evaluation of prevailing conditions, presenting a significant challenge for relevant stakeholders. Regular ecological assessments are essential for monitoring habitat health, species populations, and levels of anthropogenic pressure (Hughes et al. 2019; Leclère et al. 2020; Maxwell et al. 2020). Evaluating both current and historical conditions provides a comprehensive understanding of changes in forest area, serving as a key indicator of forest degradation. This evaluation offers a scientific basis for the necessity of further assessing the extent and implications of forest degradation. Additionally, analyzing 5- or 10-year predictions of land-use change trends under scenarios with and without implemented programs would provide valuable insights. While this study did not address such predictive modeling, it could serve as a foundation for future research to evaluate the long-term effectiveness of the applied strategies.

## Conclusion

The increase in encroachment following the designation of an area as a national park has become a fundamental challenge to the sustainability of protected areas. In the case of the KSNP, the rising trend of encroachment is largely due to the lack of intervention from relevant stakeholders. Most land conversions involve the establishment of agricultural fields, predominantly planted with robusta coffee and cinnamon. We classified encroachers into six categories: indigenous landless, indigenous people with economic opportunities, sly opportunists, indigenous people as investors, workers/profit-sharing partners, and local migrants.

This classification highlights the diverse statuses and roles of the KSNP’s land-use dynamics, with each category presenting unique implications for resource management and conservation strategies. These categories provide a crucial framework for understanding the multifaceted interactions of encroachers in the KSNP. Effective solutions must combine legal enforcement, economic alternatives (e.g., agroforestry), and clearly defined zoning within the park. Without targeted interventions, generalized solutions risk undermining efforts to reduce encroachment and safeguard national parks. Further research is necessary to model land-use change predictions under scenarios with and without program intervention to provide insights into the long-term impacts of the implemented strategies.

## Supplementary information

Appendix A

## Data Availability

Data of this research will be available from the corresponding author on reasonable request.
