# Supplementary material for: Understanding encroachment typologies through remote sensing and socio-economic analysis: enhancing national park management in Kerinci Seblat National Park, Indonesia
Source: Environ Manage. 2025 Jul 11;75(10):2763–76. doi: 10.1007/s00267-025-02218-x (PMC12457501; doi:10.1007/s00267-025-02218-x)
Supplement: Supplementary file 1 — Appendix A [file 267_2025_2218_MOESM1_ESM.docx]

**Appendix A. Accuracy Assessment and Validation Metrics**

The following tables summarize the validation results of the land use/land cover classification. Accuracy metrics are calculated based on a stratified reference dataset using expert visual interpretation of high-resolution imagery (Google Earth Pro, 2022–2023).

**Table A1. Sample allocation by land use/land cover (LULC) class based on proportional area within the RPT Sub-Watershed. The stratified sampling design ensures fair representation of all classes, including those with smaller area coverage, following best practice in accuracy assessment.**

| **LULC Class ID** | **LULC Class Name** | **Area (ha)** | **Area Proportion (%)** | **Sample Allocation (Min 10)** |
| --- | --- | --- | --- | --- |
| 1 | Bare Land | 1,097.64 | 3.41 | 22 |
| 4 | Forest | 13,489.47 | 41.9 | 161 |
| 7 | Paddy Field | 335.43 | 1.04 | 14 |
| 8 | Grass | 382.41 | 1.19 | 14 |
| 9 | Seasonal Crops | 465.21 | 1.45 | 15 |
| 10 | Annual Crops | 14,452.11 | 44.89 | 172 |
| 11 | Bush and Shrubs | 1,923.75 | 5.98 | 32 |
| 12 | Settlement | 13.14 | 0.04 | 10 |
| 13 | Water Body | 32.67 | 0.1 | 10 |
| Total | All Classes | 32,191.83 | 100 | 450 |


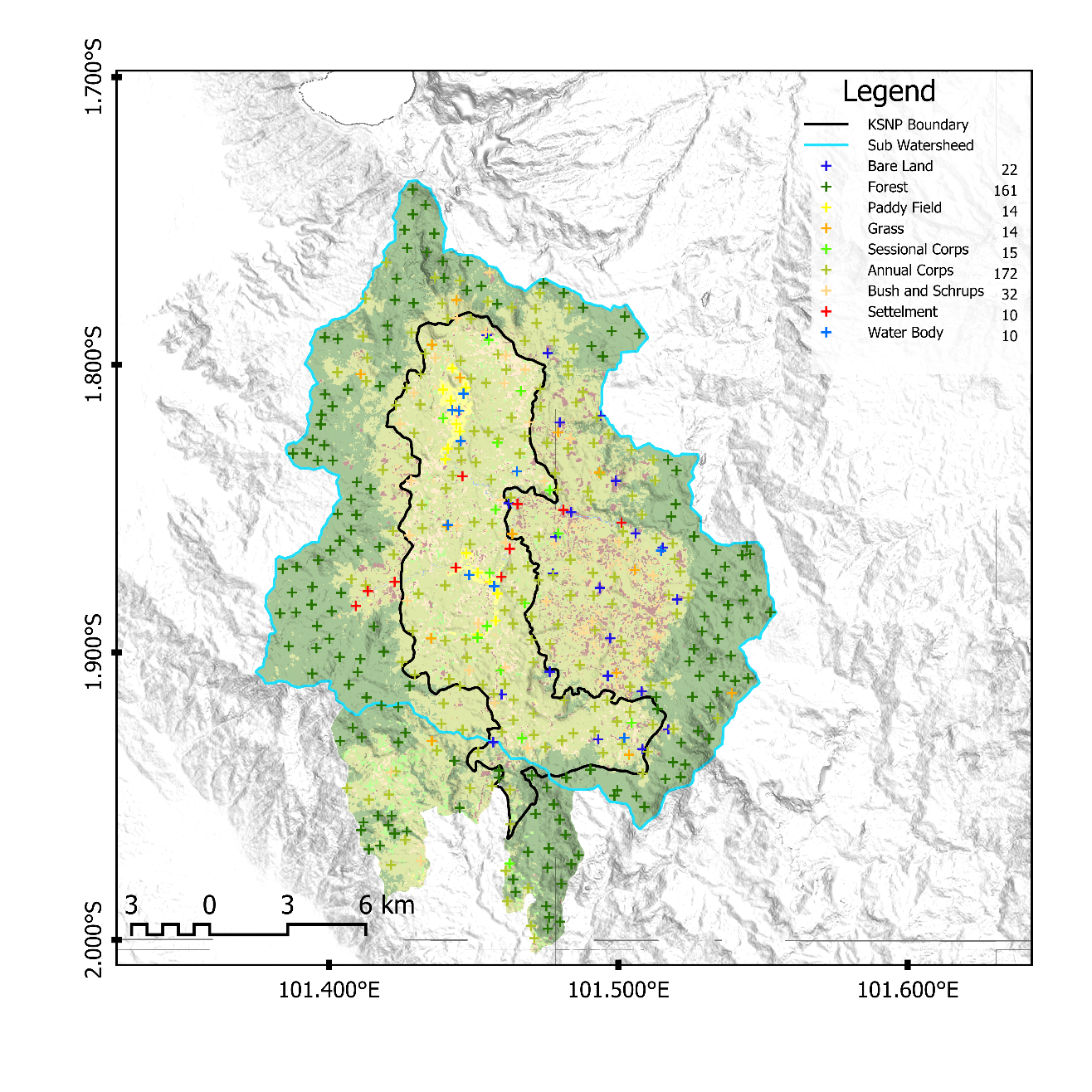


**Figure A1. Spatial distribution of 450 validation sample points over the LULC 2022 classification within the RPT Sub-Watershed area. Each colored cross symbol represents a specific land use/land cover (LULC) class with corresponding sample count displayed in the legend. The map also shows the boundary of Kerinci Seblat National Park (KSNP) in black and the delineated sub-watershed boundary in cyan.**

**Table A2. Confusion matrix comparing the classified LULC map with reference data across all sample points. Values represent the number of points per class intersection, supporting the accuracy evaluation.**

| Reference / Predicted | Pred Bare Land | Pred Forest | Pred Paddy Field | Pred Grass | Pred Seasonal Crops | Pred Annual Crops | Pred Bush and Shrubs | Pred Water Body | Pred Settlement |
| --- | --- | --- | --- | --- | --- | --- | --- | --- | --- |
| Ref Bare Land | 18 | 0 | 0 | 0 | 0 | 0 | 0 | 0 | 0 |
| Ref Forest | 0 | 159 | 0 | 0 | 0 | 0 | 2 | 0 | 0 |
| Ref Paddy Field | 0 | 0 | 11 | 0 | 0 | 0 | 0 | 0 | 0 |
| Ref Grass | 0 | 0 | 0 | 14 | 0 | 0 | 0 | 0 | 0 |
| Ref Seasonal Crops | 3 | 0 | 3 | 0 | 15 | 0 | 0 | 0 | 0 |
| Ref Annual Crops | 1 | 2 | 0 | 0 | 0 | 172 | 0 | 0 | 0 |
| Ref Bush and Shrubs | 0 | 0 | 0 | 0 | 0 | 0 | 30 | 0 | 0 |
| Ref Water Body | 0 | 0 | 0 | 0 | 0 | 0 | 0 | 10 | 0 |
| Ref Settlement | 0 | 0 | 0 | 0 | 0 | 0 | 0 | 0 | 10 |

**Table A3. Producer's accuracy indicates the probability that a reference class has been correctly classified. User's accuracy shows the probability that a classified pixel on the map actually represents that class on the ground.**

| Class | Producer's Accuracy (PA) | User's Accuracy (UA) | Total Reference Points | Correctly Classified |
| --- | --- | --- | --- | --- |
| Bare Land | 1.0 | 0.8182 | 18 | 18 |
| Forest | 0.9876 | 0.9876 | 161 | 159 |
| Paddy Field | 1.0 | 0.7857 | 11 | 11 |
| Grass | 1.0 | 1.0 | 14 | 14 |
| Seasonal Crops | 0.7143 | 1.0 | 21 | 15 |
| Annual Crops | 0.9829 | 1.0 | 175 | 172 |
| Bush and Shrubs | 1.0 | 0.9375 | 30 | 30 |
| Water Body | 1.0 | 1.0 | 10 | 10 |
| Settlement | 1.0 | 1.0 | 10 | 10 |

**Table A4. Overall accuracy represents the proportion of correctly classified samples across all classes. The Kappa coefficient measures the agreement between classification and reference data, adjusted for chance agreement.**

| Metric | Value |
| --- | --- |
| Overall Accuracy | 97.56% |
| Kappa Coefficient | 0.9657 |

Accuracy metrics were calculated based on (Congalton, 1991; Olofsson et al., 2014), while the Kappa statistic follows the formulation by (Cohen, 1960), as follows:
- Producer’s Accuracy (PA) = xᵢᵢ / ∑xᵢⱼ (row total)
- User’s Accuracy (UA) = xᵢᵢ / ∑xⱼᵢ (column total)
- Overall Accuracy (OA) = ∑xᵢᵢ / total samples
- The Kappa coefficient was calculated following (Cohen, 1960)
Where xᵢᵢ is the number of correctly classified samples in class i, and xᵢⱼ represents sample counts in the confusion matrix.
